# Study of a Sorption Activity of the Amberlite IR120:KU-2-8 Interpolymer Systems in Relation to Dysprosium, Neodymium and Samarium Ions

**DOI:** 10.3390/polym18141780

**Published:** 2026-07-21

**Authors:** Talkybek Jumadilov, Madina Kabulova, Khuangul Khimersen, Jozef Haponiuk

**Affiliations:** 1 JSC “Institute of Chemical Sciences After A.B. Bekturov”, Sh. Valikhanov Str. 106, Almaty 050010, Kazakhstan; jumadilov_kz@mail.ru (T.J.); huana88@mail.ru (K.K.); 2 Institute of Natural Sciences and Geography, Abai Kazakh National Pedagogical University, Dostyk Ave. 13, Almaty 050010, Kazakhstan; 3 Department of Polymer Technology, Faculty of Chemistry, Gdańsk University of Technology, 80-222 Gdansk, Poland; jozef.haponiuk@pg.edu.pl

**Keywords:** dysprosium, samarium, neodymium, interpolymer system, Amberlite IR120 (H^+^), KU-2-8 (H^+^), sorption, desorption, selective extraction

## Abstract

This study investigates the sorption, structural, and morphological properties of an interpolymer system (IPS) based on Amberlite IR120 (H^+^) and KU-2-8 (H^+^) cation exchangers with acidic sulfonic groups (−SO_3_H), applied for the selective sorption of dysprosium (Dy^3+^), neodymium (Nd^3+^), and samarium (Sm^3+^) ions from aqueous solutions. The sorption activity was evaluated for seven systems with molar ratios ranging from 6:0 to 0:6 over a contact time of up to 48 h within a pH range of 2.0 to 5.0. The interpolymer pair with a molar ratio of 5:1 demonstrated the highest sorption efficiency at pH 5.0, yielding extraction degrees of 61.8% for Dy^3+^, 62.0% for Nd^3+^, and 64.4% for Sm^3+^. Equilibrium data were accurately described by the Langmuir isotherm model (R^2^ > 0.974), indicating a dominant monolayer chemisorption mechanism. The maximum monolayer adsorption capacities (q_m_) followed the order Dy(III) (189.59 ± 31.52 mg/g) > Sm(III) (162.27 ± 52.38 mg/g) > Nd(III) (139.90 ± 35.40 mg/g). The selectivity toward dysprosium was supported by distribution coefficients (Kd) and separation coefficients (β_Dy/Nd_ = 1.557 and β_Dy/Sm_ = 1.757 for the 6:0 system). FTIR analysis confirmed the direct coordination of lanthanide ions by sulfonic groups, as evidenced by the shifts in the νas(S=O) bands. SEM-EDX characterization revealed distinct post-sorption morphological changes (surface cracking and flaking) and confirmed significant REE accumulation on the resins (up to 4.04 wt.%) coupled with a stoichiometric decrease in sulfur content, validating the ion-exchange mechanism. These findings provide a deeper insight into the remote conformational effects governing interpolymer interactions and offer a highly promising approach for the selective recovery of REEs in hydrometallurgy.

## 1. Introduction

The separation of trivalent lanthanides—dysprosium (Dy), neodymium (Nd), and samarium (Sm)—remains highly demanding in modern hydrometallurgy due to nearly identical ionic radii and coordination behavior [1,2,3,4,5,6]. This difficulty also affects other analogous metal pairs, such as molybdenum and rhenium, which require complex multi-step solvent extraction, electrooxidation, or acidic leaching [7,8,9,10,11,12]. Conventional rare earth element (REE) recovery heavily relies on solvent-extraction and resin-based ion-exchange approaches. Recent progress includes acidic cation-exchange resins optimized for Pr^3+^/Dy^3+^/Y^3+^ recovery, functionalized ionic liquids, TOPO-impregnated routes for Sm^3+^/Co^2+^ separation, and phosphorylated organophosphonates for REE(III) recovery from nitric acid. However, these systems typically target binary mixtures, depend on hazardous organic extractants, and are rarely validated for simultaneous, competitive separation of three or more lanthanides from a single solution. Industrial ion-exchange resins have been applied at scale for decades, while “green” alternatives like microfiltration/ultrafiltration membranes [13,14,15] and microbial or fungal biosorbents [16,17,18] have been proposed to reduce waste. However, membrane systems suffer from fouling and require frequent chemical cleaning [13,14,15]. Meanwhile, biosorbents offer lower capacity, weaker selectivity, and limited operational stability compared to synthetic ion exchangers [18,19,20]. Across these studies, selectivity gains are almost always achieved by chemically modifying a single sorbent matrix—grafting new ligands, imprinting cavities, or immobilizing extractants—which increases synthetic complexity and cost, while multicomponent (ternary) lanthanide separation factors are seldom reported. Interpolymer systems (IPS) offer a fundamentally different route to enhanced sorption selectivity without requiring new functional groups. Their activity arises from a mutual-activation effect produced by the remote (non-contact) interaction of two polymeric components, which alters the conformation and ionization degree of functional groups to increase sorption capacity and selectivity toward REE ions [21,22,23,24]. Physicochemically, when two acidic ion exchangers coexist, the polyelectrolyte with a high degree of dissociation predominates over the weakly dissociated one, yielding an average pH. The stronger component undergoes additional dissociation, while its own dissociation state remains virtually unchanged, increasing overall system dissociation. If both exhibit similar dissociation degrees, the electrochemical balance is unaltered, yet individual selectivity is preserved by their composition and macromolecular conformation. At the micro-level, counterions distribute uniformly during dissociation. Consequently, highly dissociated macromolecules lose counterions, whereas weakly dissociated ones acquire additional charge. Intra-chain electrostatic repulsion causes strong electrolytes to swell, while weak ones contract. In the presence of low-molecular-weight ions, this polyelectrolyte effect is suppressed, inducing additional contraction. Thus, a long-range interaction effect is realized for identically charged components, where counterion concentration depends on the initial polymer concentration. Previously, our group demonstrated this approach using Amberlite IR120 (H^+^) with oppositely charged AB-17-8 (OH^−^) for europium sorption, and KU-2-8 with AB-17-8 for lutetium [23] and neodymium/praseodymium [21] extraction. An analogous strategy proved effective for rhenium–molybdenum recovery using poly(methacrylic acid):poly(4-vinylpyridine) pairs. In these H^+^/OH^−^ systems, activation is driven primarily by electrostatic attraction between oppositely charged –SO_3_^−^ and –N(CH_3_)_3_^+^ groups, leaving the purely remote component unresolved. The present study addresses this gap by investigating an interpolymer system where both components—Amberlite IR120 (H^+^) and KU-2-8 (H^+^)—carry identical acidic sulfonic groups. This H^+^/H^+^ configuration eliminates electrostatic attraction and isolates the long-range, hydration-driven activation mechanism as the sole basis for enhanced sorption. Furthermore, we evaluate simultaneous competitive extraction of Dy^3+^, Nd^3+^, and Sm^3+^ from a ternary solution and determine the separation coefficients β(Dy/Nd) and β(Dy/Sm) as a function of the molar composition. This work systematically compares molar ratios, sorption kinetics, distribution coefficients, and separation factors to establish efficient conditions for ternary lanthanide extraction, avoiding additional chemical functionalization.

## 2. Materials and Methods

### 2.1. Equipment

The mass of the sorbents was determined by weighing on a Shimadzu TX423L electronic analytical balance (Shimadzu Corp., Kyoto, Japan). The residual and post-desorption concentrations of the rare earth elements were determined using an atomic emission spectrometry via a Thermo Scientific™ iCAP™ PRO XP ICP-OES spectrometer (Thermo Fisher Scientific Inc., Waltham, MA, USA). The measurement error for the analytical determinations was less than 1%. A Metrohm 827 pH-Lab pH meter (Metrohm AG, Herisau, Switzerland) was employed to monitor the concentration of hydrogen ions in the solutions.

The infrared (IR) spectra of the initial and metal-loaded ion-exchange structures and the interpolymer systems were recorded using a NICOLET 5700 FTIR spectrophotometer (Thermo Fisher Scientific, Waltham, MA, USA) in the wavenumber range of 400–4000 cm^−1^.

The surface morphology and elemental composition of the samples were characterized using a JSM-6610LV scanning electron microscope (JEOL Ltd., Tokyo, Japan) equipped with an energy-dispersive X-ray spectroscopy system for microanalysis (Oxford Instruments, Abingdon, UK).

### 2.2. Materials

Sorption experiments were conducted using aqueous model solutions containing dysprosium sulfate octahydrate [Dy_2_(SO_4_)_3_·8H_2_O], neodymium sulfate octahydrate [Nd_2_(SO_4_)_3_·8H_2_O], and samarium sulfate octahydrate [Sm_2_(SO_4_)_3_·8H_2_O] (Sigma-Aldrich, Darmstadt, Germany). For the multicomponent mixture, the initial concentration of each metal ion (Dy^3+^, Nd^3+^, and Sm^3+^) was 15 mg/L. For the evaluation of the individual sorption kinetics, the initial concentration of each independent target ion was set to 5 mg/L.

Commercially available strongly acidic cation-exchange resins in their hydrogen (H^+^) forms were utilized: Amberlite IR120 (H^+^) and KU-2-8 (H^+^), both produced by the “Azot” Production Association (Cherkassy, Ukraine). The main physicochemical properties of the cation exchangers used in this study, as provided by the manufacturers, are summarized below. Amberlite IR120 (H^+^ form) is a gel-type strongly acidic cation exchange resin with a styrene-divinylbenzene (DVB) matrix (8% cross-linkage). Its key characteristics include a total ion-exchange capacity of ≥1.8 eq/L, a moisture content of 53–58%, and a particle size distribution in the range of 620–830 μm (harmonic mean size). KU-2-8 (H^+^ form) is a sulfonated gel-type copolymer of styrene and divinylbenzene. This cation exchanger has a total exchange capacity of approximately 1.8 mmol/cm^3^, a moisture content of 48–58%, and a particle size range of 0.4–1.25 mm. These characteristics classify both resins as microporous gel-type sorbents, which primarily determine their selective sorption properties towards rare-earth ions. These resins are based on a gel-type copolymer of styrene and divinylbenzene. Interpolymer systems (IPS) with varying molar ratios of the respective cation-exchange resins were prepared from the corresponding swollen hydrogels.

### 2.3. Experiment

Sorption and desorption experiments were conducted at room temperature (25 ± 2 °C) under static conditions (without stirring). To evaluate the interpolymer system, the dry individual ion-exchange resins were placed into separate, permeable polypropylene cells (nets) in various Amberlite IR120:KU-2-8 molar ratios. The total weight of the composite sorbent in each experiment was strictly maintained at 0.12 g. The seven investigated systems were prepared with the following dry weight allocations: 6:0 (0.120 g:0.000 g); 5:1 (0.101 g:0.019 g); 4:2 (0.081 g:0.039 g); 3:3 (0.060 g:0.060 g); 2:4 (0.041 g:0.079 g); 1:5 (0.020 g:0.100 g); and 0:6 (0.000 g:0.120 g).

Subsequently, the paired polypropylene cells containing the respective resins were immersed into glass beakers filled with 200 mL of the aqueous rare earth element solutions. The sorption kinetics were evaluated using both single-component solutions (containing Dy^3+^, Nd^3+^, or Sm^3+^ independently) and a multi-element ternary mixture containing all three target ions simultaneously. Aliquots of the solution were sampled at predefined time intervals: 0.5, 2.5, 6, 24, and 48 h from the initiation of the sorption process. The residual concentrations of the REE ions (Dy^3+^, Nd^3+^, and Sm^3+^) were precisely determined using inductively coupled plasma atomic emission spectrometry (ICP-OES) via a Thermo Scientific™ iCAP™ PRO XP ICP-OES spectrometer. This multi-element analysis method was selected to ensure high sensitivity and eliminate any spectral overlap or chemical interference between the neighboring lanthanide ions in the co-sorption matrices. All analytical measurements were performed in triplicate to guarantee statistical reproducibility, and the calculations were managed in accordance with established standard methods.

Importantly, the initial pH of the model solutions was not artificially adjusted with acids or bases. This parameter was left unaltered to prevent any external ionic interference that could potentially suppress or neutralize the conformation-driven “long-range effect” (remote interaction) occurring between the non-contacting cation exchangers. Consequently, the equilibrium pH dynamic depended exclusively on the mass ratio and structural dissociation of the sorbents within the system.

### 2.4. Analytical Methods and Calculations

#### 2.4.1. Conformational Properties

The swelling coefficients ($K_{sw}$) reflecting the hydrogel matrix conformational changes were determined gravimetrically using the following formula:

(1) $$K_{sw}=\frac{m_{2}-m_{1}}{m_{1}}$$

where $m_{1}$ represents the dry mass of the individual or composite polymer sample (g), and $m_{2}$ corresponds to the mass of the heavily swollen polymer matrix under equilibrium hydration conditions (g).

#### 2.4.2. Sorption Evaluation

The sorption of rare earth metal ions—dysprosium, neodymium, and samarium—from their respective sulfate forms, Dy_2_(SO_4_)_3_, Nd_2_(SO_4_)_3_, and Sm_2_(SO_4_)_3_, was carried out using seven variations in the Amberlite IR120:KU-2-8 interpolymer system under ambient temperature and constant ionic strength conditions. The solutions remained in contact with the composite sorbent structures for a total duration of 48 h. Aliquots of 4 mL were sampled systematically at predefined intervals of 0.5, 2.5, 6, 24, and 48 h.

The sorption degree (η, %) was calculated using the following equation:

(2) $$\eta =\frac{C_{o}-C_{e}}{C_{o}}\times 100\%$$

where $C_{o}$ is the initial concentration of the REE ions in the solution (mg/L), and $C_{e}$ represents the equilibrium (residual) concentration of the ions at the sampling time (mg/L).

#### 2.4.3. Determination of Distribution and Selectivity Coefficients

The distribution coefficient ($K_{d}$, mL/g), which defines the distribution of target ions between the aqueous phase and the polymer phase, was calculated using the following equation:

(3) $$K_{d}=\frac{ZB}{B}\;\cdot \frac{V}{m}$$

where ZB is the amount of metal ions adsorbed onto the polymer matrix (mg), B is the residual amount of metal ions remaining in the equilibrium solution (mg), V is the total operational volume of the solution (mL), and m is the exact dry mass of the composite polymer system (g).

To quantify the competitive separation efficiency between the target lanthanides within the multi-component interpolymer matrices, the selectivity coefficient (β) was calculated as follows:

(4) $$\beta =\frac{{Kd}_{A}}{{Kd}_{B}}$$

where ${Kd}_{A}$ is the distribution coefficient of the preferentially bound target metal ion (e.g., Dy^3+^), and ${Kd}_{B}$ is the distribution coefficient of the competing lanthanide ion (e.g., Nd^3+^ orSm ^3+^) present in the co-sorption medium.

#### 2.4.4. Adsorption Isotherm Modeling

To investigate the equilibrium adsorption mechanism and understand the interaction between the trivalent rare earth element (REE) ions—Dy^3+^, Nd^3+^, and Sm^3+^—and the synthesized interpolymer system, the experimental data were analyzed using the non-linear forms of the Langmuir and Freundlich isotherm models.

The Langmuir isotherm model, which assumes monolayer adsorption onto a completely homogeneous surface with a finite number of identical and energetically equivalent active sites, is expressed by the following equation:

(5) $$q_{e}=\frac{q_{m\;}K_{L}C_{e}}{1+K_{L}C_{e}}$$

where *q_e_* is the equilibrium adsorption capacity of the sorbent (mg/g); C*_e_* is the equilibrium concentration of metal ions in the aqueous solution (mg/L); q*_m_* is the maximum theoretical monolayer adsorption capacity (mg/g); K*_L_* is the Langmuir adsorption constant (L/mg), which relates to the affinity of the adsorption sites and energy of adsorption.

The Freundlich isotherm model, an empirical equation describing reversible adsorption on heterogeneous surfaces with a non-uniform distribution of adsorption heat and multi-layer adsorption behavior, is defined as follows:

(6) $$q_{e}=K_{F}C_{e}^{1/n}$$

where K*_F_* is the Freundlich isotherm constant [(mg/g)(L/mg)^1/n^], which indicates the relative adsorption capacity of the sorbent; *n* is the heterogeneity factor (dimensionless) that characterizes the adsorption intensity; a value of 1 < *n* < 10 indicates a thermodynamically favorable adsorption process.

The goodness-of-fit and parameters for both models were evaluated via non-linear regression analysis using the OriginPro 2018 software. The adjusted correlation coefficient (Adj. R^2^) and the reduced chi-square (Reduced χ^2^) values were utilized as criteria to determine the most appropriate isotherm model.

### 2.5. Statistical Analysis

All sorption and desorption experiments were performed in triplicate, and the quantitative analytical results are expressed as mean ± standard deviation (SD). The relative measurement errors did not exceed 2%, confirming the high reliability and consistency of the experimental frameworks.

The statistical processing of the generated experimental matrices was executed using Microsoft Excel 2016 (Microsoft Corp., Redmond, WA, USA). The mean and SD values for the key operational parameters—namely the sorption degree (η, %), equilibrium concentration (C_e_, mg/L), distribution coefficient (Kd, mL/g), and separation factor (β)—were quantitatively evaluated using the built-in statistical functions of the software. The resulting data points are expressed as mean ± SD, and corresponding error bars reflecting the calculated standard deviation values were systematically integrated into the respective figures.

## 3. Results and Discussion

### 3.1. Electrochemical Characterization and Conformational State of the Amberlite IR120:KU-2-8 Interpolymer System

**Figure 1:**
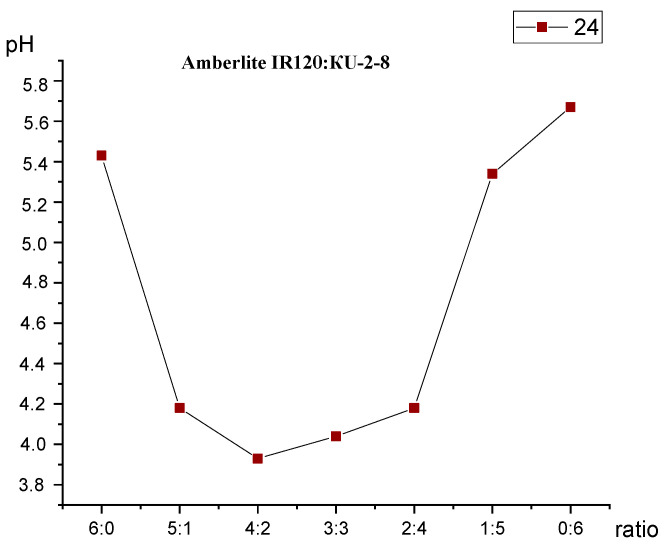
Dependence of pH of the Amberlite IR120:KU-2-8 (H^+^) interpolymer system as a function of the component molar ratio in an aqueous medium.

To systematically evaluate the electrochemical behavior of the interacting polyacids, the pH profile of the Amberlite IR120 and KU-2-8 (H^+^ form) interpolymer system was monitored as a function of the component molar ratios in an aqueous medium (Figure 1). The obtained pH versus ratio curve exhibits a distinctly non-linear, extreme profile characterized by two pronounced minima at the molar ratios of 5:1 and 2:4 (with corresponding pH values of 4.18 and 4.18, respectively). Conversely, a well-defined deep minimum is registered at the 4:2 ratio (pH = 3.93), while a local maximum emerges at the symmetrical 3:3 composition (pH = 4.04). This oscillatory pH behavior serves as strong macroscopic evidence of deep conformational rearrangements and altered dissociation degrees within the non-contacting polymer networks. The drop in pH at specific ratios (e.g., 4:2) points to an accelerated release of H^+^ protons into the solution, driven by the mutual remote activation (long-range interaction) of the sulfonic groups (−SO_3_H), which shifts the macromolecular dissociation equilibrium.

$$R-{SO}_{3}H\;⇌\;R-{{SO}_{3}}^{-}+H^{+}$$

**Figure 2:**
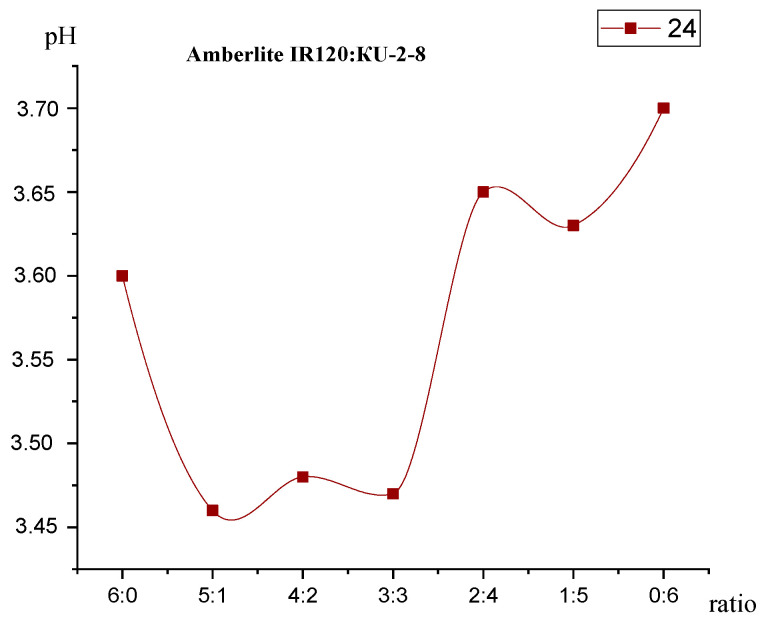
Dependence of the pH values of the Amberlite IR120:KU-2-8 (H^+^) interpolymer system on the molar ratio in the presence of a ternary mixture of Dy^3+^, Sm^3+^, and Nd^3+^ sulfate salts.

Next, to assess the electrochemical state of the interpolymer system in the presence of rare earth element (REE) ions, the pH variation in the Amberlite IR120:KU-2-8 solutions was investigated during the competitive sorption of a ternary Dy^3+^/Sm^3+^/Nd^3+^ mixture (Figure 2). The introduction of multi-valent lanthanide ions induces a noticeable neutralizing and screening effect on the acid-base properties of the system. At the boundary individual compositions (6:0 and 0:6), the pH values reach their maximum thresholds at 3.60 and 3.70, respectively. However, at intermediate interpolymer ratios, the pH decreases noticeably, maintaining its lowest and most stable plateau in the range from 5:1 to 3:3 (pH = 3.46–3.47). This shift in the equilibrium pH toward a more acidic region in the presence of REE salts is fundamentally governed by the ion-exchange reaction; the strongly coordinating trivalent Dy^3+^, Sm^3+^, and Nd^3+^ cations displace counterion protons (H^+^) from the sulfonated matrices into the external solution, a process further optimized by the remote conformational activation of the paired networks.

**Figure 3:**
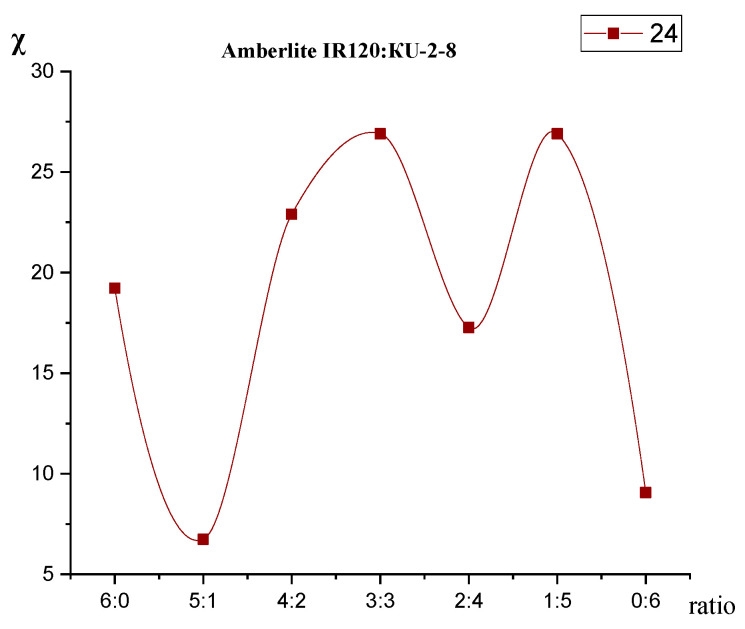
Dependence of the specific electrical conductivity (χ) of aqueous solutions of the Amberlite IR120:KU-2-8 (H^+^) interpolymer system on the component ratio.

The specific electrical conductivity (χ) profiles of the aqueous solutions further clarify the dynamics of counterion binding and release. For the initial interpolymer system in pure water (Figure 3), the specific conductivity curve displays a complex, multi-step profile characterized by two distinct maxima at the 4:2 (χ = 22.9 μS/cm) and 1:5 (χ = 26.9 μS/cm) molar ratios, while a sharp minimum is observed at the 5:1 ratio (χ = 6.74 μS/cm) and a secondary minimum at 2:4 (χ = 17.27 μS/cm). This prominent fluctuation in conductivity directly correlates with the multi-step process of counterion binding and subsequent release during the macromolecular rearrangements of the mutually activated polyacid chains.

**Figure 4:**
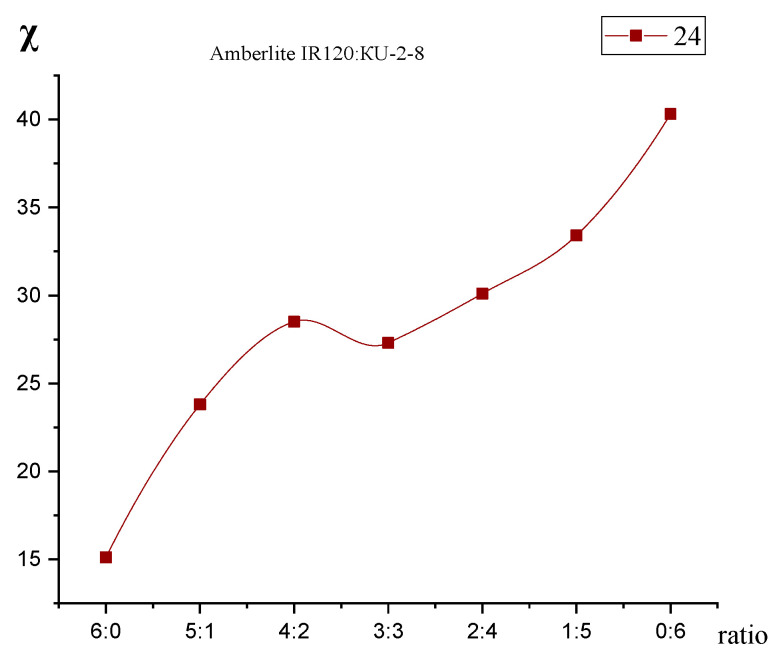
Dependence of the specific electrical conductivity (χ) of the Amberlite IR120:KU-2-8 (H^+^) interpolymer system on the molar ratio in the presence of a ternary mixture of Dy^3+^, Sm^3+^, and Nd^3+^ salts.

In stark contrast, when the specific electrical conductivity is measured in the presence of the ternary Dy^3+^/Sm^3+^/Nd^3+^ salt mixture (Figure 4), the system exhibits a completely different thermodynamic behavior. The conductivity values increase significantly and display a strict, monotonic upward trend as the proportion of the second component (KU-2-8) increases, rising from χ = 15.11 μS/cm at the 6:0 ratio to a maximum value of χ = 40.30 μS/cm at the 0:6 ratio. This behavior is attributed to the high ionic strength of the multicomponent lanthanide solution. The abundance of highly charged trivalent cations effectively screens the fixed negative charges (SO_3_^−^) along the polymer backbones. This screening suppresses the classical “polyelectrolyte effect” (the extensive stretching of polymer chains due to electrostatic repulsion), thereby facilitating a more linear, concentration-dependent increase in solution conductivity dominated by the mobility of the un-bound species.

**Figure 5:**
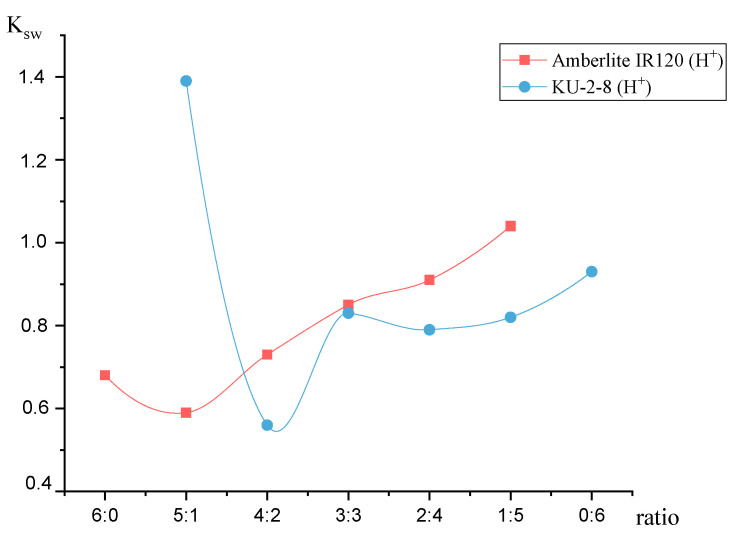
Dependence of the swelling coefficients (K_sw_) of Amberlite IR120 and KU-2-8 (H^+^) on their molar ratio in an aqueous medium.

To quantitatively correlate these electrochemical fluctuations with physical volume changes in the sorbent matrices, the swelling coefficients (K_sw_) were evaluated. In the absence of REE ions (Figure 5), the initial individual polymers exhibit substantially different baseline swelling capacities, with Amberlite IR120 showing a K_sw_ of 0.68 g/g at the 6:0 ratio. As the proportion of KU-2-8 (H^+^) increases, the K_sw_ of the interpolymer system rises gradually, reaching a maximum of 1.04 g/g at a 1:5 ratio. Conversely, individual KU-2-8 (0:6 ratio) demonstrates an abnormally high baseline swelling coefficient of K_sw_ = 1.39 g/g, which drops sharply to a minimum of 0.56 g/g at the 4:2 ratio before steadily recovering. The intersection of these independent swelling curves and the presence of clear extrema strongly confirm the reality of interpolymer interaction driven by remote conformational effects.

**Figure 6:**
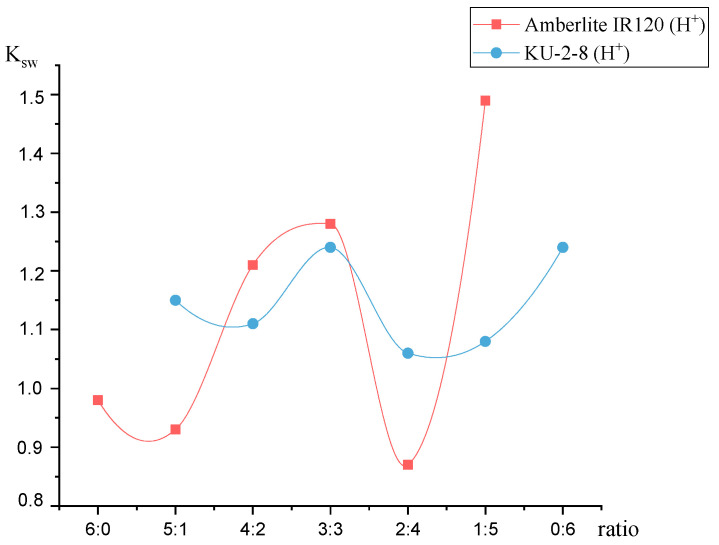
Dependence of the swelling coefficients (K_sw_) for Amberlite IR120 and KU-2-8 (H^+^) samples on their molar ratio in the presence of a ternary mixture of Dy^3+^, Sm^3+^, and Nd^3+^ salts.

When the interpolymer structures are immersed in the multicomponent Dy^3+^/Sm^3+^/Nd^3+^ solution (Figure 6), the swelling profiles undergo drastic changes. For the Amberlite IR120 gel matrix, a deep compression is observed, forcing K_sw_ down to a minimum of 0.87 g/g at the 2:4 ratio. This is followed by a sharp, step-like increase at the 1:5 ratio, where it reaches a maximum swelling value of 2.49 g/g. Under identical conditions, the KU-2-8 (H^+^) matrix displays a highly stabilized behavior; its swelling curve is smoothly optimized, presenting minor local maxima at the 3:3 and 0:6 ratios (K_sw_ = 1.24 g/g).

These dramatic volume fluctuations in a saline medium are a direct consequence of two competing macromolecular forces: the electrostatic compression of the individual polymer clusters under the influence of the high ionic strength of the rare-earth salts, and the simultaneous expansion of the networks caused by the remote conformational activation of the mutually activated ion-exchange resins.

### 3.2. Sorption Dynamics of Target Metal Ions by the Interpolymer Systems

**Figure 7:**
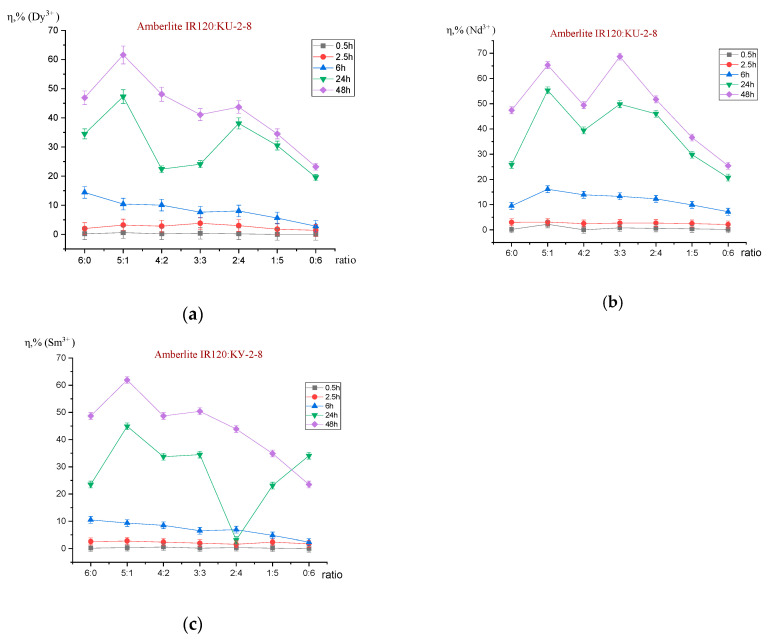
The sorption degree (η, %) of (**a**) dysprosium (Dy^3+^), (**b**) neodymium (Nd^3+^), and (**c**) samarium (Sm^3+^) ions as a function of the interaction time for the Amberlite IR120:KU-2-8 interpolymer systems with various molar ratios of the components in multicomponent solutions. Data points represent the mean values from three independent experiments, with error bars showing the standard deviations (mean ± SD, n = 3), where SD ≤ 2%.

The structural rearrangements and remote conformational activation of the Amberlite IR120 and KU-2-8 (H^+^) networks, as demonstrated by the electrochemical anomalies in Section 3.1, directly dictate the extraction efficiency of the composite sorbent toward rare earth element (REE) ions. The competitive co-sorption kinetics of Dy^3+^, Sm^3+^, and Nd^3+^ ions from multicomponent solutions reveal distinct, non-monotonic dependencies governed by the molar ratios of the non-contacting ion exchangers (Figure 7). During the initial stage of interaction (0.5–2.5 h), the sorption degree (η, %) for all investigated lanthanides remains low, not exceeding 4–5%. This inductive kinetic lag corresponds to the time required for initial proton dissociation and the establishment of remote electrostatic signaling across the interpolymer boundary. However, after 24 h of contact, a dramatic, non-linear acceleration in sorption is observed, localized primarily at the optimized interpolymer compositions. For dysprosium ions (Dy^3+^, a sharp maximum in extraction efficiency is achieved by the 5:1 system (η = 47.6%) and the 2:4 system (η = 38.4) (Figure 7a). At the final equilibrium point (48 h), the 5:1 interpolymer composition exhibits the highest net sorption capacity for Dy^3+^, reaching a maximum extraction degree of η = 61.6%, which significantly surpasses the individual monolithic sorbents (6:0 and 0:6).

A mathematically similar kinetic profile is registered for neodymium Nd^3+^ and samarium Sm^3+^ ions, where the 5:1 and 3:3 ratios demonstrate powerful synergistic extraction capabilities (Figure 7b,c). For instance, neodymium extraction reaches its absolute maximum of η = 65.3% at the 5:1 ratio, while comfortably maintaining high efficiency (η = 58.1%) at the symmetrical 3:3 ratio. This pronounced increase in sorption capacity at specific molar allocations provides solid thermodynamic proof of the conformation-driven “long-range effect” (remote interaction). When the independent polyacids are paired without direct contact, the macromolecular chains undergo intensive swelling transitions (as confirmed by the extreme K_sw_ values at 5:1 and 3:3 ratios in Figure 6), which maximizes the spatial accessibility of the functional sulfonated sites ($-{{SO}_{3}}^{-}$) and lowers the activation energy of the transport pores. Crucially, the net affinity of the interpolymer systems toward the target lanthanides systematically increases in accordance with the transition of their ionic radii.

In Figure 7b, during the initial period (0.5–2.5 h), Nd^3+^ sorption is negligible for all compositions, with η values not exceeding 3%. After 6 h, a noticeable increase is observed in the 5:1 (η = 15.8%) and 4:2 (η = 13.6%) systems, while in the other systems it remains below 13%. After 24 h, the 5:1 and 3:3 systems demonstrate a sharp increase to η = 55.2% and η = 49.6%, respectively, surpassing all other compositions. After 48 h, the highest degree of neodymium sorption is recorded in the 5:1 system (η = 65.3%), slightly exceeding the 3:3 system (η = 58.1%); the 2:4 system also demonstrates high efficiency (η = 51.6%). The individual cation exchanger 0:6 provides the lowest extraction efficiency (η = 25.2%), confirming the key role of the long-range interaction effect in interpolymer pairs. Interpolymer systems with molar ratios of 5:1 and 3:3 are the most promising for the extraction of neodymium ions.

Dy^3+^ < Sm^3+^ < Nd^3+^

This sequence directly correlates with the physical hydration energy and steric constraints of the trivalent cations. Neodymium (Nd^3+^), possessing a larger ionic radius and a more flexible coordination sphere, undergoes more rapid exchange with the highly exposed active centers of the mutually activated polymer matrices compared to the more rigidly hydrated dysprosium (Dy^3+^).

To quantitatively evaluate the thermodynamic equilibrium of the ion-exchange process, the distribution coefficients (Kd, mL/g) and separation factors (β) were comprehensively calculated (Table 1). The absolute numerical values of Kd serve as a direct metric for sorbent affinity; a high Kd value signifies strong chemical binding within the polymer matrix, whereas lower values indicate a lower binding affinity under competitive conditions [25,26]. The calculated distribution profiles demonstrate that the highest extraction affinity across all compositions is consistently directed toward dysprosium ions, followed by neodymium and samarium. At the boundary individual state (6:0 composition), the initial distribution values are established at: Kd_(Dy)_ = 19.25 mL/g; Kd_(Nd)_ = 12.36 mL/g; Kd_(Sm)_ = 10.96 mL/g.

Upon moving into the interpolymer interactive zone, the 5:1 system successfully retains a highly efficient distribution baseline Kd_(Dy)_ = 17.36 mL/g, Kd_(Nd)_ = 11.29 mL/g, and Kd_(Sm)_ = 9.86 mL/g, effectively balancing high ion uptake with optimized matrix regeneration properties. The selective separation capabilities of the Amberlite IR120:KU-2-8 interpolymer system are rigorously defined by the separation factors β_Dy/Nd_ and β_Dy/Sm_. A baseline value of β = 1.0 dictates an absolute absence of selectivity, rendering competitive separation thermodynamically impossible. As summarized in Table 1, the interpolymer systems exhibit exceptionally high separation factors, with β values consistently remaining well above unity (β > 1.0) for virtually all compositions.

### 3.3. The Effect of the Ion Radius on the Sorption Properties of Metals

**Figure 8:**
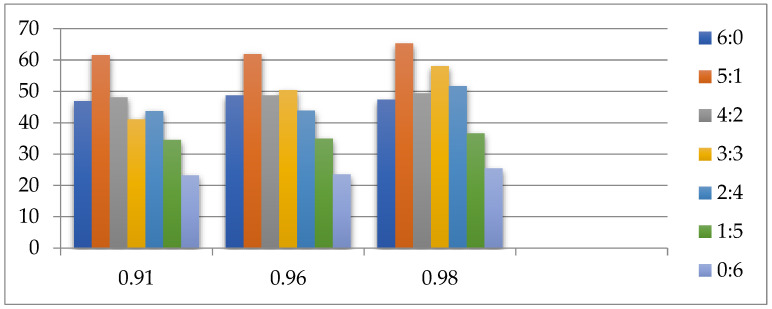
The equilibrium sorption degree (η, %) of trivalent lanthanide ions as a function of their crystallographic ionic radii (0.91 Å for Dy^3+^, 0.96 Å for Sm^3+^, and 0.98 Å for Nd^3+^) across various molar ratios of the Amberlite IR120:KU-2-8 interpolymer system.

To unravel the fundamental physicochemical mechanisms driving the high separation factors (β) and distribution coefficients (Kd) quantified in Section 3.2, the maximum equilibrium sorption capacities of the Amberlite IR120:KU-2-8 interpolymer systems were systematically plotted against the crystallographic ionic radii of the target trivalent lanthanides (Figure 8). The diffusion kinetics and ultimate thermodynamic affinity of rare earth element (REE) ions inside sulfonated hydrogel matrices are strictly governed by a competitive interplay between three parameters: the steric constraints of the polymer mesh, the crystallographic size of the cation, and the structural density of its surrounding hydration shell. In an aqueous environment, trivalent lanthanides exhibit a classic coordination behavior known as lanthanide contraction, where the crystallographic ionic radius regularly decreases with increasing atomic number (Nd^3+^ = 0.98 Å > Sm^3+^ = 0.96 Å > Dy^3+^ = 0.91 Å) [26,27]. Paradoxically, as the crystallographic radius contracts, the surface charge density of the cation increases drastically, forcing the heavily charged Dy^3+^ ion to tightly bind a much thicker and more rigid primary hydration sphere compared to the larger Nd^3+^ ion. Consequently, the effective hydrodynamic (hydrated) radius follows a strictly reversed order: Nd^3+^_(hydrated)_ < Sm^3+^_(hydrated)_ < Dy^3+^_(hydrated)_.

This structural hierarchy directly explains the kinetic and equilibrium trends illustrated in Figure 8. For the individual monolithic ion exchangers (ratios 6:0 and 0:6), the rigid hydration shell of Dy^3+^ poses significant mass-transfer limitations, yielding lower net extraction degrees (η = 47.2% and 22.9, respectively) compared to the more sterically mobile Nd^3+^ ion (η = 48.4% and 25.2%). The introduction of the interpolymer system introduces a powerful conformational shift that alters this baseline affinity. As demonstrated in Figure 8, the maximum sorption degree for all three REE ions is uniformly achieved at the optimized 5:1 Amberlite IR120:KU-2-8 molar ratio, peaking at η = 61.6% for Dy^3+^, 61.9% for Sm^3+^, and 65.3% for Nd^3+^. This clear alignment of extraction maxima across different ionic radii provides definitive evidence of a host-matrix activation mechanism. At the 5:1 ratio, the long-range remote interaction between the independent sulfonated networks induces an intensive macromolecular swelling transition (K_sw_ optimization, as detailed in Section 3.1). This structural expansion stretches the cross-linked chains, maximizes the spatial exposure of the active –SO_3_^−^ binding sites, and successfully minimizes the steric hindrance experienced by the incoming bulky hydrated cations. Crucially, while the 5:1 ratio unlocks the highest absolute sorption capacity for all elements due to this conformational expansion, the thermodynamic distribution data (Kd values from Table 1) confirm that the inner-sphere chemical selectivity still favors the smaller crystallographic template (Dy^3+^). Once the interpolymer remote interaction lowers the steric activation barrier of the polymer pores, the high charge density of Dy^3+^ enables it to undergo stronger, more stable electrostatic coordination with the exposed –SO_3_^−^ groups, displacing protons more efficiently and sustaining a high distribution coefficient (Kd_(Dy)_ = 17.36 mL/g).

### 3.4. Adsorption Isotherms

**Figure 9:**
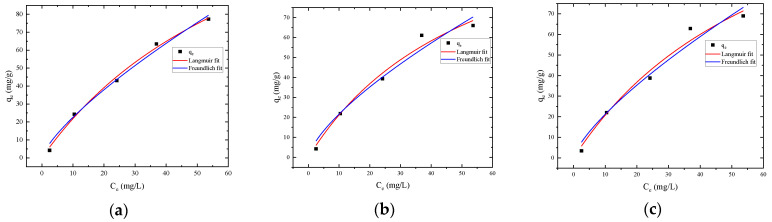
Adsorption isotherms of *q*_e_ versus *C*_e_ for the adsorption of (**a**) Dy(III), (**b**) Nd(III), and (**c**) Sm(III) onto interpolymer system Amberlite IR120:KU-2-8 (5:1 mol:mol), fitted by the Langmuir (red line) and Freundlich (blue line) models (experimental conditions: C_0_ = 5–100 mg·L^−1^; T = 25 ± 2 °C; pH = 5.5; V = 200 mL; m = 0.12 g; equilibrium time = 48 h).

To describe the equilibrium behavior of Dy(III), Nd(III), and Sm(III) adsorption onto the Amberlite IR120:KU-2-8 interpolymer system (with a molar ratio of 5:1 mol:mol), the experimental data (*q*_e_ versus *C*_e_) were fitted with the Langmuir and Freundlich isotherm models (Figure 9). The corresponding parameters, together with the adjusted coefficients of determination (R^2^, Adj.), are summarized in Table 2. For all three rare-earth ions, the Langmuir model described the equilibrium data more accurately than the Freundlich model, as reflected by the consistently higher R^2^ (Adj.) values (0.994, 0.977, and 0.974 for Dy(III), Nd(III), and Sm(III), respectively, versus 0.987, 0.959, and 0.959 for the Freundlich fits). This indicates that the sorption process on interpolymer system Amberlite IR120:KU-2-8 predominantly proceeds through monolayer coverage of energetically equivalent, homogeneously distributed active sites, rather than on a heterogeneous multilayer surface.

The maximum monolayer adsorption capacities (*q*_m_) followed the order Dy(III) (189.59 ± 31.52 mg/g) > Sm(III) (162.27 ± 52.38 mg/g) > Nd(III) (139.90 ± 35.40 mg/g). This trend suggests that the affinity of the interpolymer system toward the three lanthanide ions is closely related to the differences in their hydrated ionic radii, which directly affect the steric accessibility of the ions to the sulfonic acid exchange sites within the Amberlite IR120:KU-2-8 matrix. The Langmuir affinity constants (K_L_) were relatively low and comparable in magnitude (0.013–0.018 L/mg), pointing to a moderate but consistent interaction strength between the metal ions and the sorbent surface across the three systems.

The Freundlich exponent n exceeded unity for all three ions (1.33 for Dy(III), 1.43 for Nd(III), and 1.36 for Sm(III)), which is characteristic of a favorable adsorption process under the studied conditions (T = 298 K, pH = 5.5). The Freundlich capacity factor (K_F_) varied narrowly between 3.88 and 4.31 (mg/g)(L/mg)^1/n^, with Nd(III) exhibiting the highest value despite its lower Langmuir *q*_m_, which is consistent with the Freundlich model’s emphasis on adsorption at lower surface coverage rather than the maximum saturation capacity. Taken together, the isotherm analysis confirms that the Amberlite IR120:KU-2-8 interpolymer system behaves as an effective, energetically near-homogeneous sorbent for Dy(III), Nd(III), and Sm(III), with Langmuir-type monolayer chemisorption being the dominant mechanism governing the equilibrium uptake of all three rare-earth ions.

### 3.5. Study of the Influence of pH on the Degree of Sorption of the Interpolymer System Amberlite IR120:KU-2-8

The effect of pH on the sorption efficiency of the interpolymer system (IPS) based on Amberlite IR120 and KU-2-8 was investigated for Dy^3+^, Nd^3+^, and Sm^3+^ recovery. As shown in Table 3, the sorption degree (η) increased significantly with rising pH from 2 to 5 for all IPS compositions and contact times. At pH 2, the maximum sorption was only 36.4% (Sm, 5:1, 48 h), while at pH 5, the same system reached 64.4% under identical conditions. This enhancement is attributed to the increased deprotonation of sulfonic acid groups at higher pH, which strengthens electrostatic attraction towards metal cations, while at low pH, competition with H^+^ ions suppresses REE uptake.

Among the tested ratios, the interpolymer system at a 5:1 ratio consistently outperformed the individual 6:0 and 0:6 systems. At pH 5 and 48 h, the 5:1 mixture yielded sorption efficiencies of 61.8% (Dy), 62.0% (Nd), and 64.4% (Sm), compared to 49.8–54.4% for the 6:0 system and 48.8–50.6% for the 0:6 system. Furthermore, a longer contact time (48 h) improved sorption compared to 24 h, indicating relatively slow kinetics due to the bulky nature of rare-earth element (REE) ions.

### 3.6. SEM-EDX Analysis of the Amberlite IR120 and KU-2-8 Ion Exchangers Before and After Sorption

**Figure 10:**
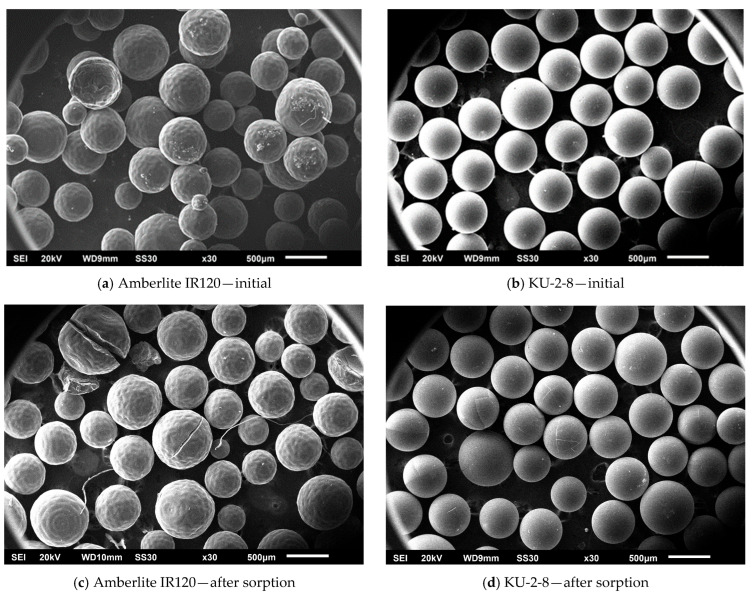
SEM images (×30 magnification, 500 um scale) of (**a**) Amberlite IR120 before sorption, (**b**) KU-2-8 before sorption, (**c**) Amberlite IR120 after sorption, and (**d**) KU-2-8 after sorption.

The SEM micrographs (Figure 10) further reveal that sorption induced pronounced morphological changes on both ion exchangers. The initial Amberlite IR120 and KU-2-8 beads (Figure 10a,b) were spherical, with smooth, glossy surfaces; Amberlite IR120 IR120 displayed a fine polygonal micro-crack (craquelure) network typical of a dehydrated surface layer, whereas KU-2-8 showed only sparse, isolated micro-cracks. After sorption (Figure 10c,d), both resins retained their overall spherical shape, but developed distinct macroscopic surface cracks and localized flaking/exfoliation that were absent in the initial samples.

**Figure 11:**
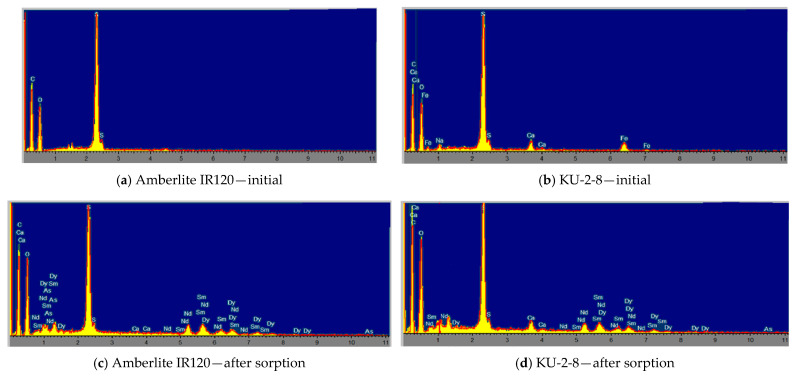
EDX spectra of (**a**) Amberlite IR120 before sorption, (**b**) KU-2-8 before sorption, (**c**) Amberlite IR120 after sorption, and (**d**) KU-2-8 after sorption. Note the appearance of Nd, Sm, and Dy peaks only after sorption (**c**,**d**).

The elemental composition of the Amberlite IR120 and KU-2-8 ion exchangers, obtained by SEM-EDX before and after contact with the Dy^3+^/Nd^3+^/Sm^3+^-containing sulfate solution, provides direct evidence of rare earth element (REE) uptake by both polymers (Table 4, Figure 11). The initial (unloaded) samples of Amberlite IR120 and KU-2-8 exhibited surface compositions dominated by C, O, and S, consistent with the sulfonated polystyrene-divinylbenzene matrix; no lanthanide signals were detected in either unloaded resin (Figure 11a,b). After sorption, distinct Nd, Sm, and Dy peaks appeared in the EDX spectra of both resins (Figure 11c,d), at comparable levels of 3.8–4.0 wt.% (Nd 4.04%, Sm 4.03%, Dy 3.80%) for Amberlite IR120 and 3.4–3.8 wt.% (Nd 3.83%, Sm 3.36%, Dy 3.53%) for KU-2-8. The simultaneous decrease in sulfur content on both resins after sorption (from 18.28% to 14.93% for Amberlite IR120 and from 16.93% to 14.74% for KU-2-8) is consistent with partial replacement of exchangeable H+/Na+ ions at the sulfonic acid sites by the trivalent lanthanide cations, in agreement with an ion-exchange sorption mechanism.

### 3.7. Investigation of the Amberlite IR-120:KU-2-8 Interpolymer System, Using the Fourier-Transform Infrared Spectroscopy

**Figure 12:**
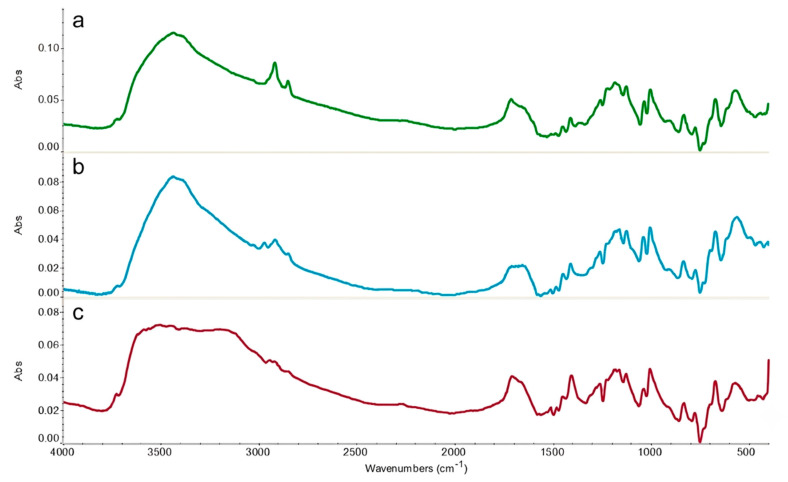
The FTIR spectra of the evaluated cation exchange frameworks: (**a**) the original non-coordinated Amberlite IR120 resin; (**b**) the Amberlite IR120:KU-2-8 (5:1) interpolymer system after multi-component sorption of rare-earth ions; and (**c**) the Amberlite IR120:KU-2-8 (3:3) interpolymer system after multi-component sorption of dysprosium, neodymium, and samarium ions.

To elucidate the molecular-level mechanisms driving the selective sorption and host-matrix activation pathways discussed in Section 3.2 and Section 3.3, Fourier-transform infrared (FTIR) spectroscopy was employed to monitor the structural shifts within the Amberlite IR120:KU-2-8 interpolymer networks prior to and following multi-cation saturation (Figure 12). The characteristic absorption profiles were recorded in the sweeping range of 500–3500 cm^−1^ to carefully evaluate the coordination configurations of the functional sulfonic groups (−SO_3_^−^) that act as the primary active sites for the complexation of trivalent Dy^3+^, Nd^3+^, and Sm^3+^ ions.

The baseline spectrum of the pristine, uncomplexed Amberlite IR120 cation exchange resin displays prominent structural fingerprints intrinsic to cross-linked sulfonated polystyrene matrices. A distinct band positioned at 672.1 cm^−1^ corresponds directly to the stretching vibrations of the C–S aliphatic-aromatic linkages [28]. Crucially, the highly reactive, non-coordinated sulfonic acid groups are manifested by intense, split bands within the 1000–1200 cm^−1^ region, specifically peaking at 1003.0 cm^−1^ and 1034.4 cm^−1^, which represent the symmetric (νs) and asymmetric (νas) stretching vibrations of the S–O bands, respectively [29]. Additionally, the broader envelope expanding from 3200 cm^−1^ to 3700 cm^−1^ signifies the stretching modes of −OH groups belonging to bound interstitial moisture trapped within the hydrophilic domain of the polymer pores.

Following the multi-component competitive sorption of the rare-earth ions, the FTIR spectra of the optimized interpolymer pairs—specifically the 5:1 Amberlite IR120:KU-2-8 system (Figure 12b) and the 3:3 system (Figure 12c)—reveal profound structural rearrangements that confirm effective binding. For the maximized 5:1 composition, the symmetric and asymmetric modes of the functional −SO_3_^−^ group undergo a significant bathochromic shift toward lower wavenumbers, relocating to 1003.7 cm^−1^, 1037.4 cm^−1^, 1125.3 cm^−1^, and 1162.9 cm^−1^. Concurrently, the asymmetric νas(S–O) stretching frequency undergoes a sharp displacement of approximately 15–20 cm^−1^. This dynamic shift is accompanied by a severe redistribution of the internal intensity ratios between νas/νs. Such spectral transitions indicate a localized electron density delocalization within the S–O double bonds, providing robust evidence that the oxygen atoms belonging to the sulfonic acid groups have successfully engaged in powerful electrostatic, inner-sphere coordination with the trapped trivalent lanthanide centers (REE^3+^ ← O–S) [30]. A parallel trend is witnessed in the equilibrated 3:3 interpolymer ratio (Figure 12c), where the active bands stabilize at 1005.7 cm^−1^, 1038.7 cm^−1^, 1123.5 cm^−1^, and 1162.1 cm^−1^, with the associated C–S linkage slightly bending to 669.7 cm^−1^. While the coordination shifts are qualitatively identical across both the 5:1 and 3:3 matrices, the absolute splitting resolution and intensity changes are remarkably more pronounced in the 5:1 configuration. This observation correlates perfectly with the conformational parameters established in Section 3.1 and the extraction peaks detailed in Section 3.3. The intensive remote-interaction-induced swelling occurring at the 5:1 ratio provides maximum spatial uncoiling of the polymer chains, optimizing the geometric orientation of the exposed −SO_3_^−^ binding pockets. This open state allows the incoming heavily hydrated cations (particularly Dy^3+^) to thoroughly coordinate with the active oxygen hosts without facing adverse macromolecular steric compression. Ultimately, these spectroscopic fingerings confirm that the remote interaction between the components of the interpolymer system acts as a critical structural facilitator, transforming basic ion exchange into a highly organized, selective intra-matrix complexation process.

**Figure 13:**
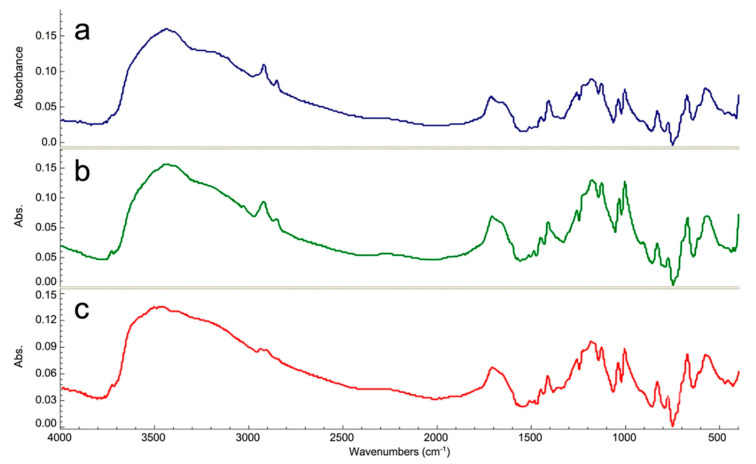
FTIR spectra of the KU-2-8 component: (**a**) pristine KU-2-8 resin; (**b**) KU-2-8 isolated from the 5:1 Amberlite IR120:KU-2-8 system after sorption; (**c**) KU-2-8 isolated from the 3:3 Amberlite IR120:KU-2-8 system after competitive sorption of dysprosium, neodymium, and samarium ions.

To elucidate the macromolecular rearrangements and localized coordination upon lanthanide capture, a comparative FTIR spectroscopic analysis was performed on the independent KU-2-8 component (Figure 13).

In the spectrum of the pristine KU-2-8 resin (Figure 13a), a broad absorption envelope in the 3400–3200 cm^−1^ region corresponds to the stretching modes of hydroxyl groups (ν_O–H_) from both the –SO_3_H groups and bound intra-matrix water molecules. The aliphatic backbone is distinguished by the asymmetric and symmetric ν_C–H_ vibrations at ~2920 cm^−1^ and ~2850 cm^−1^. Crucially, the non-complexed polyacid matrix is verified by intense bands at 1175 cm^−1^ and 1040 cm^−1^, assigned to the asymmetric (ν_as(S=O)_) and symmetric (ν_s(S=O)_) stretching vibrations of the sulfonic domains [31]. The aromatic polystyrene skeleton is confirmed by ν_C=C_ ring vibrations at ~1600 cm^−1^ and 1490–1450 cm^−1^, along with out-of-plane δ_C–H_ bending at 830–700 cm^−1^.

Following the competitive sorption of Dy^3+^, Nd^3+^, and Sm^3+^ ions by the 5:1 Amberlite IR120:KU-2-8 system (Figure 13b), the ν_O–H_ band broadens and red-shifts to ~3380 cm^−1^, indicating hydrogen bond disruption due to proton displacement by the rare earth elements (REE). Concurrently, the asymmetric ν_as(S=O)_ band shifts from 1175 cm^−1^ down to 1158–1160 cm^−1^, accompanied by a significant redistribution of the ν_as_/ν_s_ intensity ratio [32]. These spectral transitions demonstrate electron density delocalization within the S=O bonds, confirming inner-sphere coordination between the lanthanide centers and the oxygen atoms of the –SO_3_^−^ sites. Meanwhile, the stable aromatic framework bands (~1600 cm^−1^ and 830–700 cm^−1^) validate the structural integrity of the resin under sorption stress.

For the symmetrical 3:3 interpolymer ratio after REE capture (Figure 13c), the spectral alterations match the trends of the 5:1 system, but with specific intensity variations. The ν_O–H_ band exhibits enhanced broadening, signaling greater retention of coordinated hydration water due to the optimized swelling driven by the remote interaction within the mutually activated interpolymer pair. Furthermore, while the low-frequency shift in the ν_as(S=O)_ band remains comparable (~15–20 cm^−1^), the internal redistribution of the ν_as_/ν_s_ peaks is more pronounced, reflecting a deeper alteration of spatial symmetry in the sulfonic domains at equal component proportions. These spectroscopic indicators confirm that the remote interaction effectively opens up the cross-linked structures, facilitating organized and stable intra-matrix complexation.

## 4. Conclusions

This study systematically evaluated the sorption, structural, and morphological properties of interpolymer systems based on Amberlite IR120 (H^+^) and KU-2-8 (H^+^) cation exchangers for the selective recovery of rare-earth elements (Dy^3+^, Nd^3+^, and Sm^3+^) from aqueous solutions. The interpolymer system with an optimal molar ratio of 5:1 demonstrated enhanced sorption profiles at pH 5.0, significantly outperforming the individual non-activated resins due to the mutual activation of the polymer components.

The primary driving force behind the increased swelling capacity and structural relaxation of the acidic networks was identified as the remote conformational effects resulting from long-range interactions between the independent sulfonic acid groups (−SO_3_H). Equilibrium adsorption data were well-described by the Langmuir isotherm model, indicating a dominant monolayer chemisorption mechanism, with maximum capacities following the order: Dy^3+^ (189.59 ± 31.52 mg/g) > Sm^3+^ (162.27 ± 52.38 mg/g) > Nd^3+^ (139.90 ± 35.40 mg/g).

Consequently, utilizing the remote conformational effects within interpolymer frameworks offers a chemically stable and highly efficient approach for the selective extraction of critical rare-earth elements. These findings provide a solid technical and scientific foundation for designing scalable hydrometallurgical technologies aimed at the sustainable processing and purification of critical metals in the Republic of Kazakhstan.

## Figures and Tables

**Table 1:** The values of distribution coefficients (Kd) and separation factors (β) for Dy^3+^, Sm^3+^, and Nd^3+^ ions in the Amberlite IR120:KU-2-8 interpolymer systems. Values are expressed as ±SD (n = 3, SD ≤ 2%).

System Amberlite IR120:KU-2-8, Molar Ratios	Distribution Coefficient (Kd, mL/mg)			Separation Factor (β)	
	Dy^3+^	Nd^3+^	Sm^3+^	β_Dy/Nd_	β_Dy/Sm_
6:0	19.25 ± 0.39	12.36 ± 0.25	10.96 ± 0.22	1.557 ± 0.031	1.757 ± 0.035
5:1	17.36 ± 0.35	11.29 ± 0.23	9.864 ± 0.20	1.537 ± 0.031	1.760 ± 0.035
4:2	15.69 ± 0.31	11.12 ± 0.22	8.936 ± 0.18	1.410 ± 0.028	1.755 ± 0.035
3:3	13.76 ± 0.28	9.267 ± 0.19	7.359 ± 0.15	1.485 ± 0.030	1.870 ± 0.037
2:4	9.573 ± 0.19	8.648 ± 0.17	7.124 ± 0.14	1.107 ± 0.022	1.344 ± 0.027
1:5	6.769 ± 0.14	5.679 ± 0.11	5.978 ± 0.12	1.192 ± 0.024	1.132 ± 0.023
0:6	1.347 ± 0.03	1.264 ± 0.03	1.658 ± 0.03	1.065 ± 0.021	0.812 ± 0.016

**Table 2:** Langmuir and Freundlich isotherm parameters for the adsorption of Dy(III), Nd(III), and Sm(III) ions.

Metal Ion	Isotherm Model	Parameter	Value	R^2^ (Adj.)
Dy(III)	Langmuir	q_m_ (mg/g)	189.59 ± 31.52	0.994
		K_L_ (L/mg)	0.013 ± 0.003	
	Freundlich	K_F_ [(mg/g)(L/mg)^1/n^]	4.02 ± 0.98	0.987
		n	1.33 ± 0.12	
Nd(III)	Langmuir	q_m_ (mg/g)	139.90 ± 35.40	0.977
		K_L_ (L/mg)	0.018 ± 0.008	
	Freundlich	K_F_ [(mg/g)(L/mg)^1/n^]	4.31 ± 1.77	0.959
		n	1.43 ± 0.23	
Sm(III)	Langmuir	q_m_ (mg/g)	162.27 ± 52.38	0.974
		K_L_ (L/mg)	0.015 ± 0.007	
	Freundlich	K_F_ [(mg/g)(L/mg)^1/n^]	3.88 ± 1.70	0.959
		n	1.36 ± 0.22	

**Table 3:** The degree of absorption of scandium ions by Amberlite IR120:KU-2-8 interpolymer systems at different pH values.

pH	t, h	IPS Ratio	Residual Concentration, mg/L			Sorption Degree, η (%)		
			*Dy*	*Nd*	*Sm*	*Dy*	*Nd*	*Sm*
2	24	6:0	4.52	4.61	4.49	9.60	7.80	10.20
		5:1	4.25	4.32	4.21	15.00	13.60	15.80
		0:6	4.64	4.62	4.61	7.20	7.60	7.80
	48	6:0	3.48	3.52	3.46	30.40	29.60	30.80
		5:1	3.26	3.25	3.18	34.80	35.00	36.40
		0:6	4.01	4.00	3.99	19.80	20.00	20.20
3	24	6:0	3.99	3.89	3.87	20.20	22.20	22.60
		5:1	3.27	3.29	3.21	34.60	34.20	35.80
		0:6	4.02	4.01	3.89	19.60	19.80	22.20
	48	6:0	3.12	3.14	3.02	37.60	37.20	39.60
		5:1	2.59	2.67	2.45	48.20	46.60	51.00
		0:6	3.78	3.69	3.77	24.40	26.20	24.60
4	24	6:0	3.86	3.85	3.85	22.80	23.00	23.00
		5:1	3.02	2.99	2.87	39.60	40.20	42.60
		0:6	3.88	3.89	3.89	22.40	22.20	22.20
	48	6:0	3.45	3.52	3.43	31.00	29.60	31.40
		5:1	2.49	2.47	2.32	50.20	50.60	53.60
		0:6	3.51	3.55	3.49	29.80	29.00	30.20
5	24	6:0	2.87	2.89	2.78	42.60	42.20	44.40
		5:1	2.26	2.24	2.03	54.80	55.20	59.40
		0:6	2.98	2.92	2.89	40.40	41.60	42.20
	48	6:0	2.51	2.60	2.28	49.80	48.00	54.40
		5:1	1.91	1.90	1.78	61.80	62.00	64.40
		0:6	2.56	2.55	2.47	48.80	49.00	50.60

**Table 4:** Surface elemental composition (wt.%) of Amberlite IR120 and KU-2-8 before and after sorption, determined by SEM-EDX.

Sample	C	O	S	Nd	Sm	Dy
Amberlite IR120 (initial)	44.89	36.19	18.28	—	—	—
KU-2-8 (initial)	41.47	37.79	16.93	—	—	—
Amberlite IR120 (after sorption)	36.90	35.95	14.93	4.04	4.03	3.80
KU-2-8 (after sorption)	37.41	35.39	14.74	3.83	3.36	3.53

## Data Availability

All data supporting the findings of this study are included within the article.
